# A novel technique for modified all-inside repair of bucket-handle meniscus tears using standard arthroscopic portals

**DOI:** 10.1186/s13018-017-0692-y

**Published:** 2017-12-04

**Authors:** Jing Hui Yik, Bryan Thean Howe Koh, Wilson Wang

**Affiliations:** Department of Orthopaedic Surgery, National University Health Systems (NUHS), 1E Kent Ridge Road, NUHS Tower Block Level 11, Singapore, 119228 Singapore

**Keywords:** Arthroscopy, Bucket-handle meniscus tear, Novel technique

## Abstract

**Background:**

Bucket-handle meniscus tears (BHMT) are often displaced and unstable. The inside-out technique of repairing such tears is currently the gold standard. All-inside repair with meniscal fixators is getting increasingly popular. Shortcomings of the inside-out technique include neurovascular complications, especially saphenous nerve palsy, and retention of a non-resorbable suture which can result in discomfort to patient, granuloma formation, and a foci of infection. Hence, the purpose of this project was to innovate a novel all-inside technique to precisely reduce and fix BHMT while avoiding neurovascular complications and retention of a non-resorbable suture.

**Methods:**

Routine arthroscopic portals were created on a patient’s left knee with a displaced BHMT. Through the anteromedial portal, a conjoint pseudo double lumen cannula was inserted. Two limbs of a reduction suture were passed through the cannula, one over the “femoral” surface of the meniscus, one over the “tibial” surface of the meniscus anterior to the biceps femoris tendon, with the knee flexed at 20° to avoid injury to the saphenous nerve. Suture limbs were passed out percutaneously and tensioned.

**Results:**

Anatomic reduction was ensured under arthroscopic visualization with ease. All inside repair was performed using the vertical mattress suture configuration. Reduction sutures were subsequently removed by cutting flush to the skin and pulling on one suture limb. The patient was back to full activities with minimal discomfort 8 months post-operatively.

**Conclusion:**

The technique described is superior to existing techniques for the following reasons: (1) Reduction of the displaced meniscal tear is “extra-meniscal,” avoiding further trauma to a damaged meniscus. (2) Tensioning of the two suture limbs created promotes better control of reduction through tensioning. (3) Risk of discomfort, infection, and neurovascular damage caused by a retained suture is reduced. (4) No additional portals/equipment is required. We encourage this novel technique to be attempted by surgeons.

## Background

The meniscus deepens the tibial articular surface, stabilizes the knee joint, allows load transmission, reduces articular contact stress, and aids in lubrication [11]. Meniscal repairs are preferable over partial or total meniscectomies as they aim to restore a functional meniscus and possibly prevent early degenerative changes [7, 13].

Various surgical techniques have been described for repairing bucket-handle meniscus tears (BHMT): all-inside, inside-out, outside-in, and modifications of these techniques. The inside-out technique is the gold-standard and especially useful for middle third tears, posterior horn tears, and displaced BHMT. An incision is made either posteromedially or posterolaterally to access the capsule and allow sutures to be passed inside-out under arthroscopic visualization. In the setting of an unstable, displaced BHMT, inside-out repair provides accurate reduction and strong fixation of the torn meniscus [1]. The outside-in technique is most useful for anterior horn tears but can also be employed for middle third tears and BHMT. All-inside repair with meniscal fixators is increasingly popular due to ease of use, reduced operative time, and reduction in neurovascular complications [4].

However, there are limitations and drawbacks to each technique. For the purposes of this article, we will focus on BHMT, which are typically displaced and unstable. The all-inside repair with meniscal fixators may not allow satisfactory coaptation and stable fixation for a BHMT.

Hybrid repairs with initial inside-out repair to reduce and provisionally fix the bucket handle fragment before subsequent all-inside repair with meniscal fixators can overcome this problem. The inside-out repair has several disadvantages: Firstly, it necessitates an incision and dissection down to capsule, with attendant risks of infections and neurovascular complications, as high as 21% [8]. Saphenous nerve palsy is the most neurologic complication encountered. Secondly, traditional inside-out repair leaves a knot of non-resorbable suture material outside the capsule. This potentially leads to prominence of the knot, granuloma formation, and infection. Patients often complain of knot prominence as incisions are on the sides of the knee where extra-capsular tissue layers are thin. However, this may be under-reported as it may not be considered a complication per se.

The authors introduce a modification of the hybrid repair for BHMT, using an inside-out reduction suture followed by all-inside repair with meniscal fixators. This avoids the disadvantages while retaining the advantages of conventional inside-out repair.

## Case summary

**Fig. 1:**
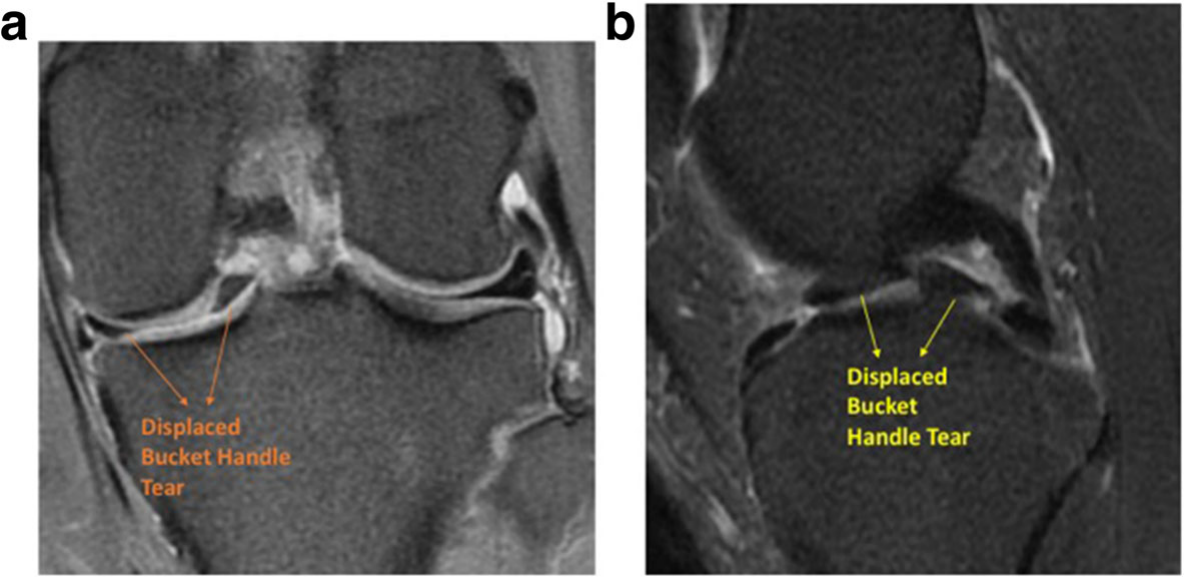
Magnetic resonance imaging of the visualized meniscus tear. **a** Coronal proton density fat suppression image of the patient’s left knee showing a displaced bucket-handle tear of the medial meniscus. **b** Sagittal T2 fat suppression image of the patient’s left knee again showing the displaced medial meniscus bucket-handle tear

A 34-year-old Chinese female with no previous notable medical or surgical history presented to clinic after sustaining an atraumatic twisting injury to the left knee while hiking. Post injury, she had pain on weight bearing, was unable to fully extend her left knee, and noted mild swelling. On physical examination, range of motion (ROM) was 0–140°, there was medial joint line tenderness, and the anterior drawer test was positive. MRI of the left knee showed a displaced bucket-handle medial meniscus tear in addition to a partial-thickness mid-substance ACL tear (Fig. 1).

The patient was subsequently counseled and consented for arthroscopic debridement and repair of the bucket-handle medial meniscus tear.

## Surgical technique

### Diagnostic arthroscopic examination

**Fig. 2:**
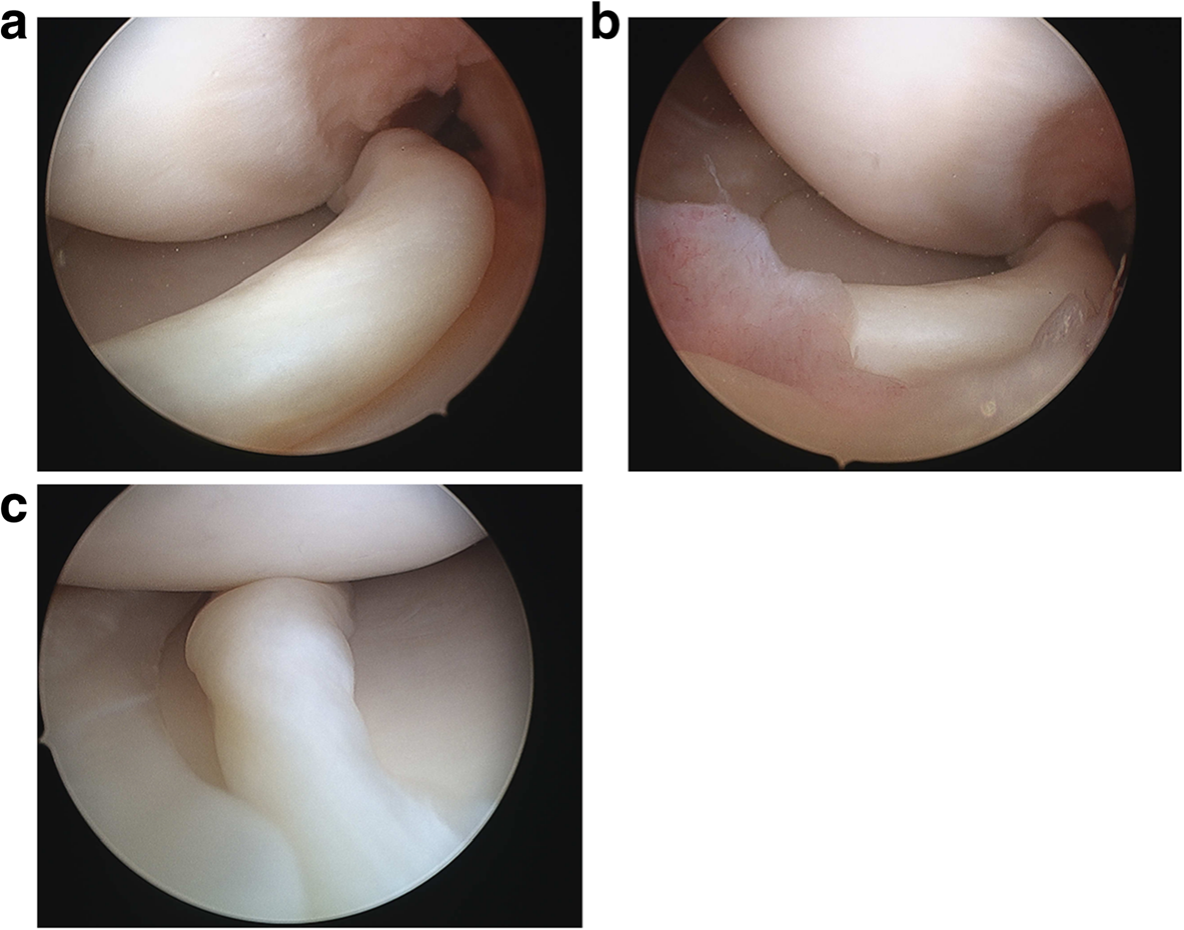
Intra-arthroscopic visualization of the bucket-handle meniscus tear. **a** and **b** show a displaced bucket-handle meniscus tear (BHMT) in this patient extending from the middle to posterior third of the meniscus. **c** shows provisional reduction of the BHMT through blunt probe manipulation

First, routine diagnostic arthroscopic examination of the knee joint was performed through standard anterolateral and anteromedial portals. In the setting of a displaced BHMT, provisional reduction was achieved through manipulation of the knee externally and manipulation of the torn meniscus fragment with an instrument such as an arthroscopic probe. The meniscus tear was assessed for size, location, chronicity, and pattern. Arthroscopic examination of the left knee confirmed a displaced bucket-handle medial meniscus tear involving the entire middle third and part of the posterior horn. The instruments used for the inside-out component was from the meniscal stitcher set for inside-out repair (Smith & Nephew), while that used for the all-inside component was from the FAST-FIX 360 Meniscal Repair System (Smith & Nephew) (Fig. 2).

### Passage of inside-out reduction sutures

**Fig. 3:**
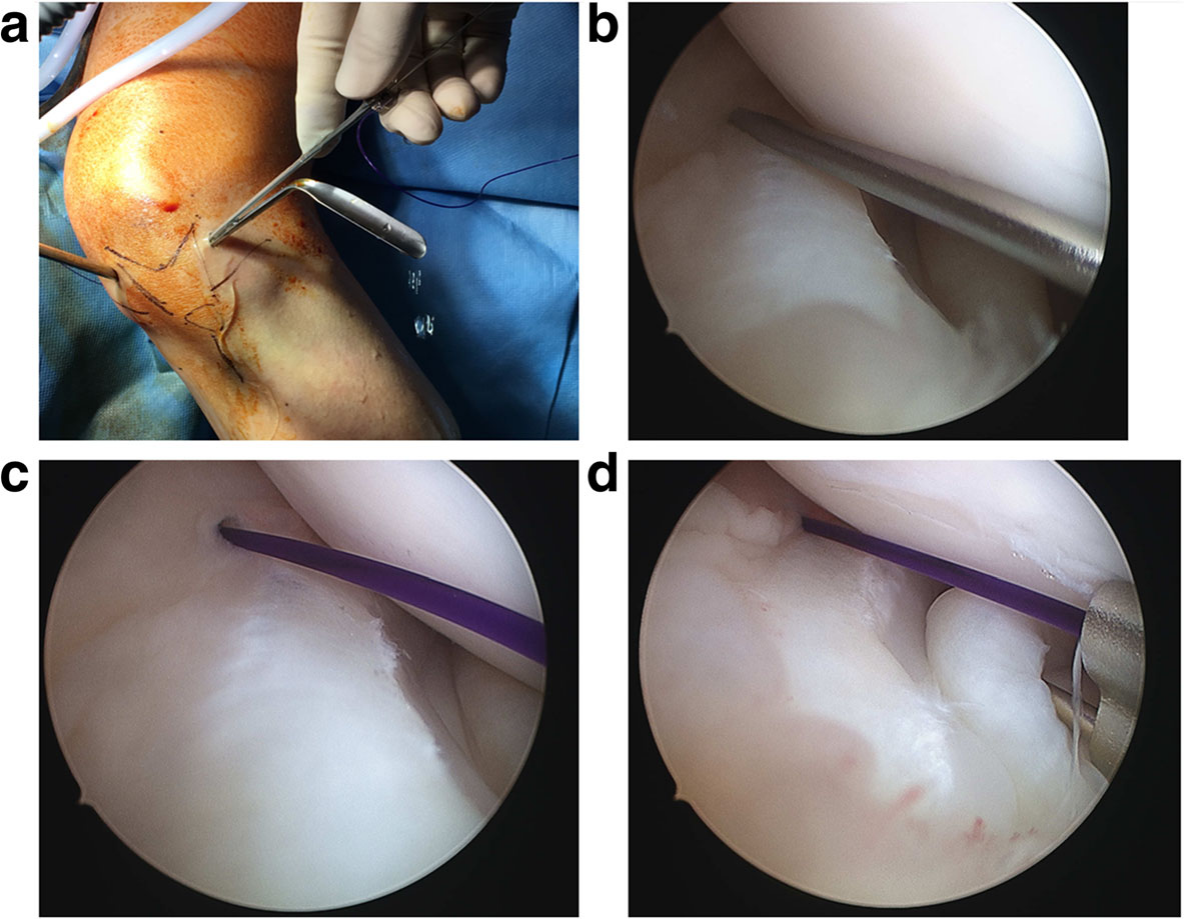
Arthroscopic images showing initial reduction of the displaced meniscus. **a** shows positioning of the double lumen cannula. **b** and **c** show passage of the needle above the femoral surface of the meniscus, with care not to traumatize the meniscal substance. **d** shows passage of the second need below the tibial surface of the meniscus

The next step involves passage of the inside-out reduction sutures above the femoral surface and below the tibial surface of the meniscus. The arthroscope was placed in the anteromedial portal, and a zone-specific double lumen cannula is inserted through the anterolateral portal. The cannula was positioned, and a flexible needle is passed through the first lumen of the cannula above the femoral surface of the meniscus. The needle then pierced out of the joint capsule with the knee in 20° of flexion. The needle has an “open end” that allows a suture to be hooked on. After passage of the “femoral” limb of the suture, the other limb of the same suture was then hooked on to the needle. This needle was then passed through the second lumen of the cannula, below the tibial surface of the meniscus and out of the joint capsule with the knee in 20° of flexion. This became the “tibial” limb of the suture. The double lumen cannula could now be withdrawn completely. The cannula used had a “conjoined” double lumen, which allowed the sequential passage of needles attached to both ends of a single suture, without subsequent obstruction to subsequent cannula withdrawal (Fig. 3).

The passage of the inside-out needles and sutures can be performed safely in a percutaneous manner for both medial and lateral meniscus tears. The critical structure to avoid on the medial side is the saphenous nerve, which emerges from the adductor canal to pierce the fascia lata between tendons of sartorius and gracilis before passing downwards on the medial side of the leg. With the knee in flexion, the saphenous nerve crosses the joint line at, or slightly behind the posteromedial corner of knee. The authors recommend passing the needle with the knee in 20° of flexion and to keep the needle anterior to the posteromedial corner of the knee. The critical structure to avoid on the lateral side is the common peroneal nerve, which is posterior to the biceps femoris tendon. The authors recommend passing the needle with the knee in 90° of flexion and to keep the needle anterior to the biceps femoris tendon, which can be easily palpated. For effective stabilization of the tear, the reduction suture should be placed at the midpoint of the BHMT, which should be anterior to either the posteromedial corner of the knee or the biceps femoris tendon, thus allowing safe passage of the inside-out needles and sutures in a percutaneous manner.

### Achieving desired reduction and stabilization of the torn meniscus

**Fig. 4:**
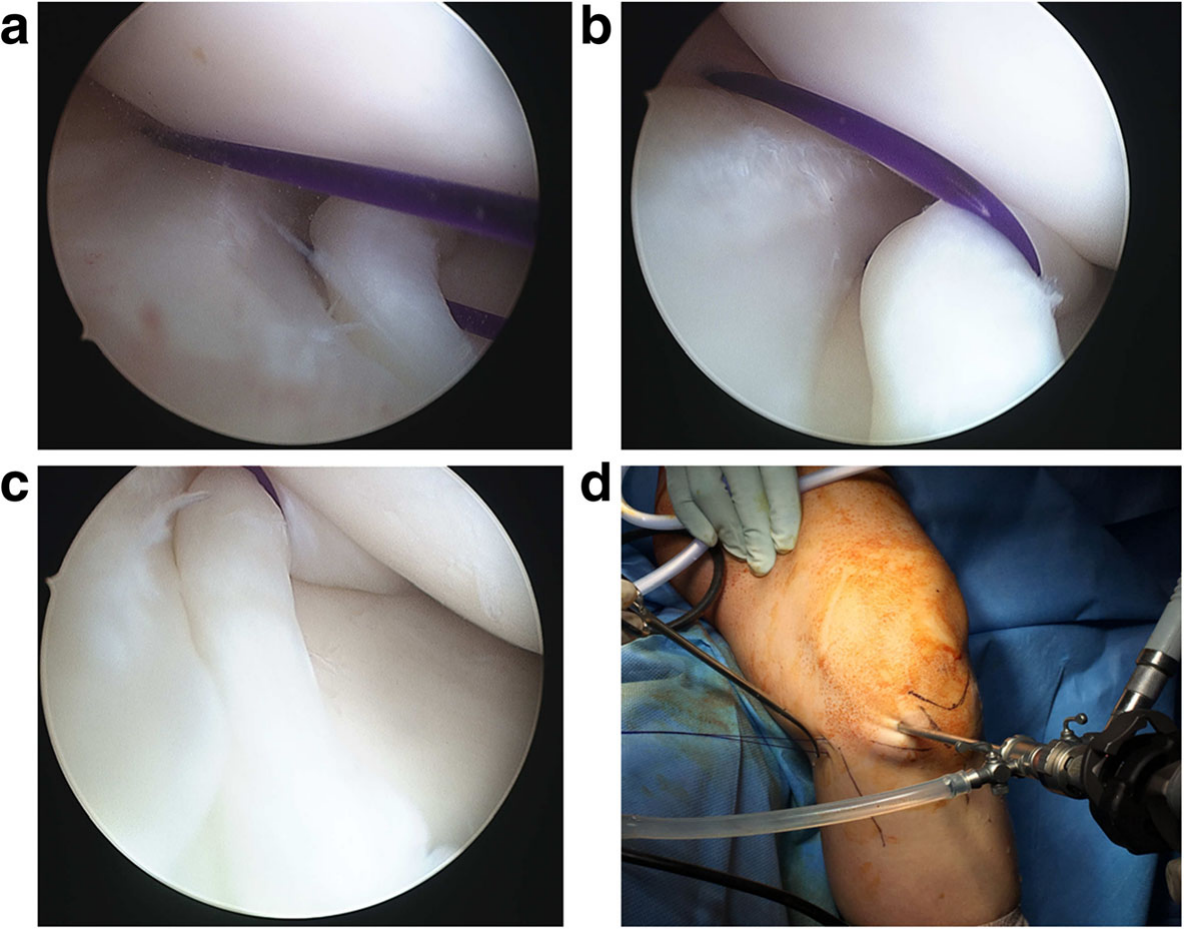
Arthroscopic images showing tensioning of the reduced meniscus. **a** shows the superior and inferior inside-out reduction sutures in position with good placement. **b** and **c** show tensioning of the reduction sutures to achieve optimal reduction. **d** shows the use of artery forceps to maintain desired tension on the two reduction suture limbs

The two limbs of the suture were then tensioned simultaneously to achieve satisfactory reduction of the torn meniscus under arthroscopic visualization. A hemostatic artery was applied on the suture limbs, flush to skin, to maintain tension and, hence, reduction of the torn meniscus. A second inside-out reduction suture can also be used to attain anatomic reduction if required, bearing in mind the safe zones for passage of the inside-out needles (Fig. 4).

### All-inside repair with meniscal fixators

**Fig. 5:**
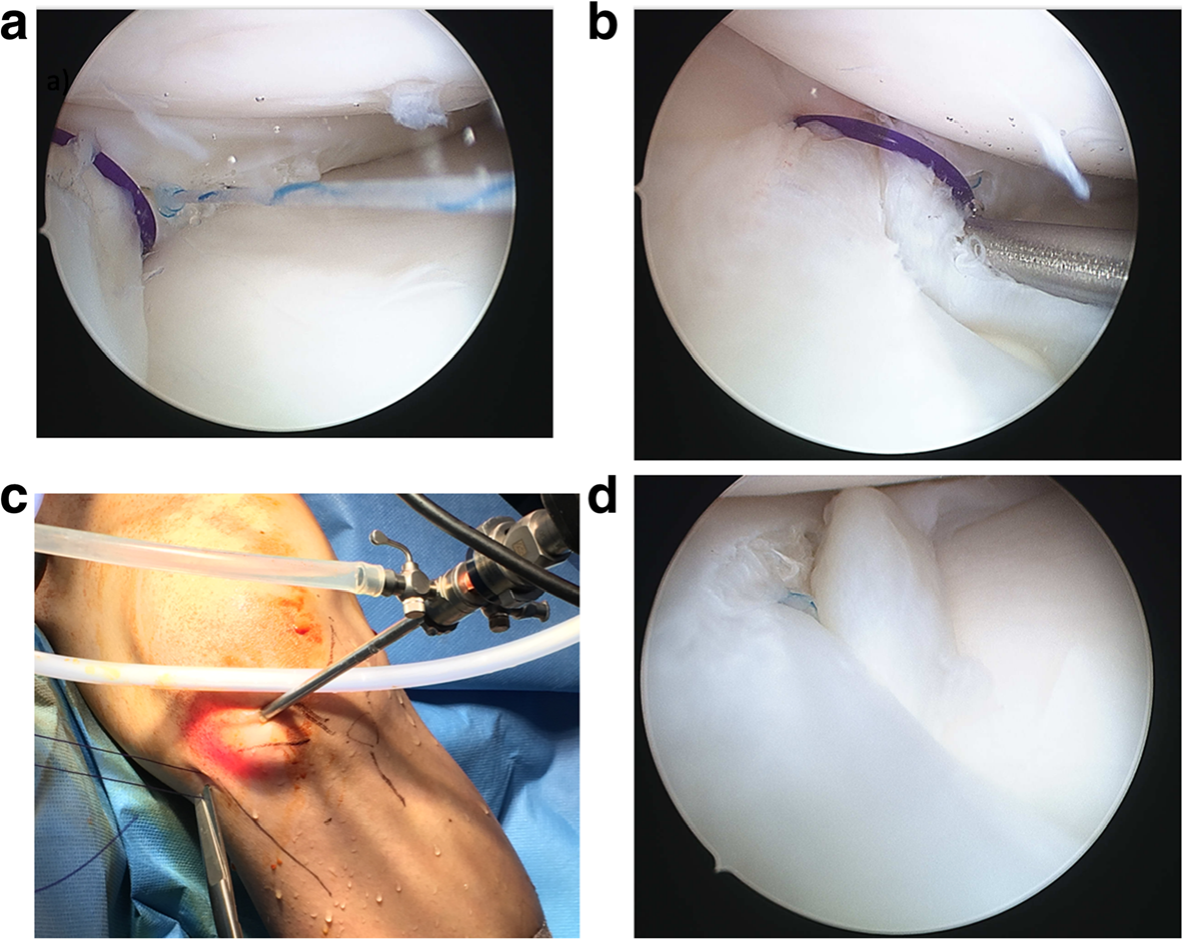
Intra-operative images showing the final repair. **a** shows passing of the all-inside meniscus fixator posterior to the reduction suture. **b** shows passing of a second all-inside meniscus fixator anterior to the reduction suture. **c** shows cutting of one limb of the reduction suture flush to skin followed by removal of the reduction suture by pulling on the other side. **d** shows a well-positioned reduction of the BHMT with two all-inside meniscus fixators after removal of the reduction sutures

**Fig. 6:**
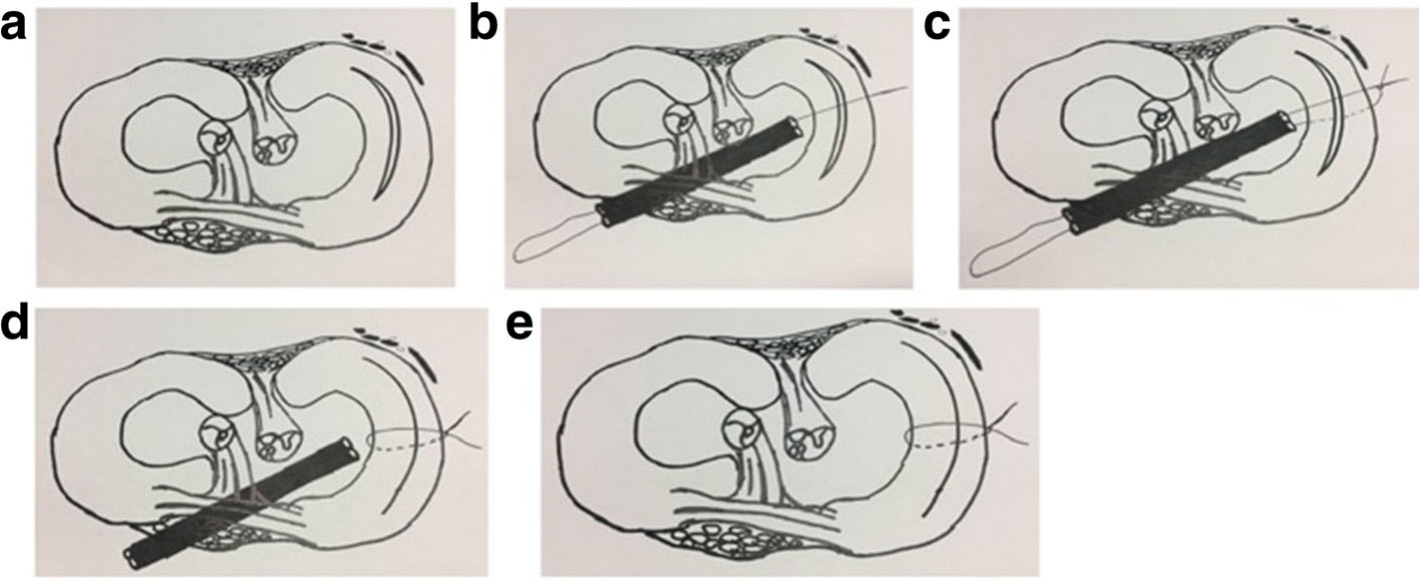
Summary of illustrations of surgical technique. **a** Illustration showing medial meniscus buckle-handle meniscus tear. **b** Passing of the superior inside-out reduction suture. **c** Passing of the inferior inside-out reduction suture and tensioning of both reduction sutures. **d** and **e** Tensioning of both reduction sutures to achieve the desired reduction

Once satisfactory reduction and stabilization of the torn meniscus has been achieved with the reduction suture in place, all-inside repair with meniscal fixators can be performed. Multiple fixators are likely to be required for a BHMT. The authors recommend repairs starting with fixators immediately anterior and posterior to the reduction suture. Each subsequent fixator should then be placed progressively further away. It is also advised to place fixators on both the femoral and tibial sides of the tear to reduce puckering of the meniscus. The all-inside repairs can be performed in either vertical mattress or horizontal mattress suture configurations, although the vertical mattress is still considered the gold standard [10]. Finally, once meniscal repair is completed, the reduction suture can be removed by cutting either suture limb flush to the skin and pulling on the other suture limb. The reduction technique that we propose is illustrated in Figs. 5 and 6.

Six months post-operatively, the patient was pain free. Range of motion of her left knee was 0–130°. The anterior drawer test demonstrated a good firm endpoint. There was mild medial joint line discomfort but no tenderness on palpation. She was back to full sporting activities after 8 months.

## Discussion

Meniscal tears are common injuries treated by orthopedic surgeons. Long-term follow-up studies have demonstrated increased arthritic changes after partial meniscectomy when compared with the anatomically normal contralateral knee [7]. The load transmitted across a knee joint increases with the amount of meniscus removed. As such, meniscal repairs are attempted in suitable patients (young; active) with suitable tears (simple longitudinal tears especially in the red-red/red-white zone) to try and restore the natural function and avoid early arthritic changes [13]. BHMT are often displaced and unstable, requiring inside-out suture repair with or without all-inside repair. The rationale for inside-out suture repair is that, unlike all-inside meniscal repair, it permits accurate reduction, stabilization, and coaptation of the tear edges [1]. The disadvantages of using an inside-out suture repair include the need for skin incisions either posterolaterally or posteromedially where there is risk of common peroneal nerve and saphenous nerve palsy, respectively. Overall risk of infections or neurovascular complications have been reported to be as high as 21% [8].

The proposed technique has several advantages over conventional inside-out repair. One feature of the proposed technique is that the inside-out reduction suture is used purely to achieve and maintain anatomic reduction of the torn meniscus while all-inside meniscus repair is done. It can be removed once meniscal repair is completed. This avoids the need for additional skin incisions because the suture does not need to be tied down onto the joint capsule, and hence, the risks of infection and neurovascular complications can be avoided. Avoiding the need for skin incisions and dissection down to capsule also potentially reduces operative time, [4] postoperative pain, and morbidity. Removing the suture after meniscus repair is completed also avoids any problems with knot prominence, granuloma, and infection that may be encountered with conventional inside-out repairs. Avoiding the need to tie sutures over the posterior capsule in this technique also avoids potential flexion contractures seen in conventional inside-out repair, which was reported by Morgan in 1991 [12].

Another feature of this technique is that the inside-out reduction suture is passed above the femoral surface and below the tibial surface of the meniscus. The position of the suture limbs above and below the meniscus, similar to a vertically oriented suture, also allows rotatory control. Tensioning of individual suture limbs may thus enable a more anatomic reduction. The conventional inside-out technique usually uses horizontal mattress sutures because vertical sutures are difficult to place; hence, it loses the rotatory control that is especially important in the case of an unstable, displaced BHMT where the torn fragment is often flipped or rotated. The technique proposed here allows rotatory control of the torn fragment through placement of the suture limbs above and below the meniscus in a vertical orientation. Ahn et al. [1] described a modified inside-out repair technique that involves passage of suture through the torn fragment to achieve rotatory control and improve reduction, but this may further damage the torn meniscus. The technique proposed here involves passing the inside-out reduction suture above and below the meniscus in an “extra-meniscal” manner, avoiding further trauma to a meniscus that is already damaged.

Advantages of all-inside repair techniques include ease of use and reduced operative time [9]. First-generation devices for all-inside meniscus repair with rigid fixators such as meniscal arrows, screw, and staples were associated with risks of implant-induced chondral damage and synovitis. Second-generation suture- and anchor-based devices were designed to minimize this risk as they leave only suture on the meniscal surface. There was also concern of inadequate fixation strength with first-generation meniscal fixators, especially for tears near the meniscocapsular junction. For this reason, Ahn had described modified all-inside suturing techniques using either posterolateral or posteromedial portals for tears near the meniscocapsular junction [2, 3]. However, recent studies have also demonstrated that second-generation suture- and anchor-based devices provide fixation comparable to the classic vertical mattress suture repair technique in terms of mean failure load and displacement after cyclic loading [5, 6]. The FAST-FIX 360 device from Smith & Nephew, used in this study, is one such device. Even when the all-inside repair is performed not vertically but horizontally (which is technically easier), the load to failure is not reduced [6]. However, in the case of an unstable and displaced BHMT, all-inside repair with either first- or second-generation devices cannot achieve satisfactory reduction or maintain that reduction. This proposed technique uses the inside-out reduction suture to achieve anatomic reduction and coaptation of the tear edges, yet avoids the disadvantages of conventional inside-out repairs. Stable fixation is then attained through all-inside meniscal repair with a second-generation suture-based device, before removal of the reduction suture. Gapping and puckering of the meniscus can be avoided by alternating the devices on both the femoral and tibial sides of the meniscus. All-inside meniscal repairs can be done in either a vertical or horizontal orientation.

This study has several limitations. Clinical outcomes for the proposed technique have not been studied, due to the relative infrequency of BHMT. Future prospective studies are warranted to study failure rates, functional outcomes, and complication rates with this technique. Also, recent systematic review comparing all-inside and inside-out meniscal repairs showed overall low levels of evidence and limited instruments for outcome measurement [8]. If subsequent high level evidence can show equivalent outcomes for all-inside and inside-out techniques, then cost effectiveness may favor inside-out repairs over all-inside repairs using meniscal fixators which tend to be expensive. However, the authors believe that most BHMT are best suited to all-inside repair as they usually extend to the posterior horn where inside-out repairs pose the risk of damage to the popliteal neurovascular bundle.

The proposed technique uses an inside-out suture to achieve and maintain anatomic reduction, followed by repair of a BHMT with second-generation all-inside meniscal fixators. To the best of the authors’ knowledge, the combined use of an inside-out suture for meniscus reduction with all-inside repair of a BHMT is not a previously described technique. It potentially avoids the risks and morbidity of conventional inside-out repair, reduces operative time, and is easy to use.

## Conclusion

The technique described is superior to existing techniques for the following reasons: (1) Reduction of the displaced meniscal tear is “extra-meniscal,” avoiding further trauma to a damaged meniscus. (2) Tensioning of the two suture limbs created promotes better control of reduction through tensioning. (3) Risk of discomfort, infection, and neurovascular damage caused by a retained suture is reduced. (4) No additional portals/equipment is required. We encourage this novel technique to be attempted by surgeons.

## Abbreviations

ACL: Anterior cruciate ligament
BHMT: Bucket-handle meniscus tear
ROM: Range of motion
